# Association between vitamin D deficiency and chronic kidney disease progression: A GRADE-based dose-response systematic review and meta-analysis

**DOI:** 10.1097/MD.0000000000050522

**Published:** 2026-09-04

**Authors:** Sayed Yousef Mojtahedi, Mohsen Sedighiyan, Paniz Pourpashang

**Affiliations:** a Pediatric Chronic Kidney Disease Research Center, Gene, Cell and Tissue Research Institute, Tehran University of Medical Sciences, Tehran, Iran; b Department of Pediatric Nephrology, School of Medicine, Bahrami Hospital, Tehran University of Medical Sciences, Tehran, Iran; c Department of Clinical Nutrition, Bahrami Children’s Hospital, Tehran University of Medical Sciences, Tehran, Iran.

**Keywords:** chronic kidney disease, CKD progression, dialysis, dose-response, end-stage renal disease, estimated glomerular filtration rate, GRADE, inflammation, meta-analysis, vitamin D deficiency

## Abstract

**Background:**

This systematic review and dose-response meta-analysis aimed to evaluate the association between serum vitamin D status and chronic kidney disease (CKD) progression.

**Methods:**

We searched PubMed, Scopus, Web of Science, and Embase for relevant observational and cohort studies published between 1990 and 2024. Eligible studies reported baseline vitamin D levels and CKD progression outcomes. We performed a random-effects meta-analysis to calculate the pooled odds ratio (OR) for CKD progression. A dose-response meta-analysis examined the relationship between serum 25-hydroxyvitamin D (25(OH)D) levels and risk, while subgroup analyses explored follow-up duration, study quality, and comorbid conditions. We assessed evidence certainty using the Grading of Recommendations Assessment, Development and Evaluation approach.

**Results:**

Eleven studies met the inclusion criteria. The pooled analysis suggested that vitamin D deficiency was associated with 33% higher odds of CKD progression (OR = 1.33, 95% confidence interval [CI]: 1.06–1.67; *P* = .019); however, between-study heterogeneity was considerable (I-squared heterogeneity statistic [*I*^2^] = 98.2%). The pooled estimate should therefore be interpreted cautiously as an average association across heterogeneous studies. A dose-response analysis of 10 studies suggested higher odds of CKD progression per 10 ng/mL lower 25(OH)D concentration (OR = 1.18, 95% CI: 1.09–1.25), although its interpretation was limited by reconstructed category-level exposure values. Subgroup analyses showed stronger associations in studies with long-term follow-up (≥5 years; OR = 2.07, 95% CI: 1.29–3.14; *P* = .001) and high-quality studies (OR = 1.35, 95% CI: 1.06–1.69; *P* = .03). The Grading of Recommendations Assessment, Development and Evaluation assessment rated the certainty of evidence as very low for the overall association and low for the dose-response analysis, primarily because of the observational study designs and substantial between-study heterogeneity.

**Conclusion:**

Lower serum 25(OH)D levels were associated with higher odds of CKD progression. However, the observational nature of the evidence does not establish that routine screening or vitamin D supplementation can prevent renal function decline. Prospective studies and randomized trials are needed to clarify the clinical utility of vitamin D assessment and supplementation for renal outcomes.

## 1. Introduction

Chronic kidney disease (CKD) is a major public health concern, affecting a significant portion of the global population.^[1]^ It is characterized by a progressive decline in kidney function, typically assessed by estimated glomerular filtration rate (eGFR) or evidence of kidney damage persisting for at least 3 months.^[2]^ The prevalence of CKD is rising due to factors such as population aging, increasing rates of diabetes and hypertension, and improved diagnostic tools.^[3]^ According to the World Health Organization, CKD is currently the 12th leading cause of death worldwide and is expected to climb in rankings in the coming decades.^[4]^

Without timely interventions, CKD can advance to end-stage renal disease (ESRD), requiring dialysis or kidney transplantation.^[5]^ Moreover, CKD is linked to serious complications, including cardiovascular disease, anemia, and mineral bone disorders, underscoring the urgency of effective management strategies.^[6]^

Recent research has emphasized the potential role of vitamin D in CKD pathophysiology. Vitamin D, a fat-soluble prohormone, is synthesized in the skin via ultraviolet light exposure and obtained from dietary sources.^[7–10]^ It is essential for calcium and phosphate homeostasis, bone health, and immune regulation. Beyond these traditional roles, vitamin D influences inflammation, immune modulation, and cellular differentiation.^[11–13]^

Observational studies suggest that vitamin D deficiency may hasten CKD progression, elevating risks of adverse outcomes such as proteinuria, kidney fibrosis, and cardiovascular events.^[14]^ Several clinical trials and cohort studies have investigated the benefits of vitamin D supplementation in slowing CKD progression and improving outcomes.^[15]^ However, results are inconsistent: some indicate protective effects from adequate vitamin D levels, while others show no significant association.^[16]^

Given these inconsistencies, a thorough synthesis of the evidence is warranted. This systematic review and meta-analysis aims to consolidate data from observational and cohort studies to elucidate the association between vitamin D deficiency and CKD progression, thereby informing clinical practice.

## 2. Methods

### 2.1. Study design

This systematic review and meta-analysis evaluated the association between vitamin D deficiency and CKD progression. We adhered to the Preferred Reporting Items for Systematic Reviews and Meta-Analyses (PRISMA) guidelines for conducting and reporting.^[17]^ The meta-analysis integrated data from observational and cohort studies examining the relationship between vitamin D deficiency and CKD progression. The protocol was registered with the International Prospective Register of Systematic Reviews (CRD42024625359) and reported in accordance with PRISMA.

### 2.2. Ethics and consent

This study was a systematic review and meta-analysis of previously published aggregate data. It did not involve direct contact with human participants or access to identifiable individual-level data; therefore, institutional ethics committee or institutional review board approval and informed consent were not required.

### 2.3. Eligibility criteria

We included only observational and cohort studies meeting predefined criteria. Participants were adults (≥18 years) diagnosed with CKD, defined by an eGFR <60 mL/min/1.73 m^2^ or kidney damage lasting at least 3 months, according to the Kidney Disease: Improving Global Outcomes guidelines.^[18]^ Studies involving patients across CKD stages (1–5) were eligible. Included studies reported baseline vitamin D levels, measured as serum 25-hydroxyvitamin D (25(OH)D) concentrations, with deficiency defined as <20 ng/mL or <50 nmol/L. We considered studies assessing the effect of vitamin D deficiency or insufficiency on CKD progression, with or without corrective interventions.

Primary outcomes were defined a priori as CKD progression, operationalized as a composite of sustained eGFR decline (≥30%–40% or rate of decline), doubling of serum creatinine, progression to ESRD, initiation of dialysis, or kidney transplantation, in accordance with the Kidney Disease: Improving Global Outcomes recommendations. Studies using proteinuria or albuminuria as the primary outcome were excluded; however, studies reporting these markers as secondary outcomes or part of a composite progression endpoint were eligible if they also provided data on the prespecified eGFR/ESRD-based measures.

We limited inclusion to peer-reviewed original articles in English. Reviews, editorials, conference abstracts without full text, letters, and non-peer-reviewed works were excluded. Studies lacking baseline vitamin D data or CKD progression outcomes, those involving non-CKD populations or acute kidney injury, and animal studies were also omitted.

### 2.4. Information sources and search strategy

A comprehensive literature search was conducted to identify relevant observational and cohort studies examining the association between vitamin D deficiency and CKD progression. We systematically searched PubMed, Scopus, Web of Science, Cochrane Library, and Embase from 1990 to November 13, 2024, with no language restrictions. The search strategy combined controlled vocabulary (Medical Subject Headings and Emtree terms) and free-text keywords related to CKD (e.g., “chronic kidney disease,” “renal impairment,” and “glomerular filtration rate”), vitamin D status (e.g., “vitamin D deficiency,” “25-hydroxyvitamin D,” and “vitamin D insufficiency”), and CKD progression outcomes (e.g., “eGFR decline,” “end-stage renal disease,” “dialysis,” and “kidney transplantation”). The strategy was adapted for each database to account for differences in indexing and was designed to capture both observational and cohort studies. Details of the complete search strings for each database are provided in File S1, Supplemental Digital Content 1.

In addition to the electronic database searches, we manually reviewed the reference lists of all included articles and relevant reviews. We also searched clinical trial registries (ClinicalTrials.gov and World Health Organization International Clinical Trials Registry Platform) for ongoing or unpublished studies. To minimize publication bias, gray literature was examined through institutional repositories, ProQuest Dissertations & Theses, conference proceedings, OpenGrey, and Google Scholar.

### 2.5. Study selection process

The study selection process followed a rigorous, systematic approach to ensure that only the most relevant studies were included in this review. Initially, the results from the electronic database searches, gray literature, and manual searches were imported into reference management software (EndNote version 10; Thomson ResearchSoft) for screening and organization. Duplicates were removed, and the remaining studies were screened in 2 stages: title and abstract screening, followed by full-text screening.

In the first stage, 2 independent reviewers (SYM and PP) screened the titles and abstracts of all identified studies against the predefined inclusion and exclusion criteria. Studies that were clearly irrelevant or did not meet the eligibility criteria were excluded at this stage. If the relevance of a study was uncertain based on the title and abstract, the study was carried forward for full-text screening. Any discrepancies between reviewers were resolved through discussion, and if necessary, a third reviewer was consulted.

After the initial title and abstract screening, the full texts of the remaining studies were retrieved and assessed for eligibility by the same 2 independent reviewers. The studies were reviewed in detail to ensure they met all the inclusion criteria, such as focusing on adult patients with CKD, reporting baseline vitamin D levels, and measuring CKD progression (e.g., changes in eGFR and progression to ESRD). Studies evaluating outcomes like proteinuria or albuminuria were excluded to reduce heterogeneity, as per the predefined exclusion criteria. In cases of disagreement between the 2 reviewers during the full-text screening, the issue was resolved by consensus or, if needed, by involving a third reviewer. The reasons for exclusion at the full-text screening stage were documented and summarized.

**Figure 1.**
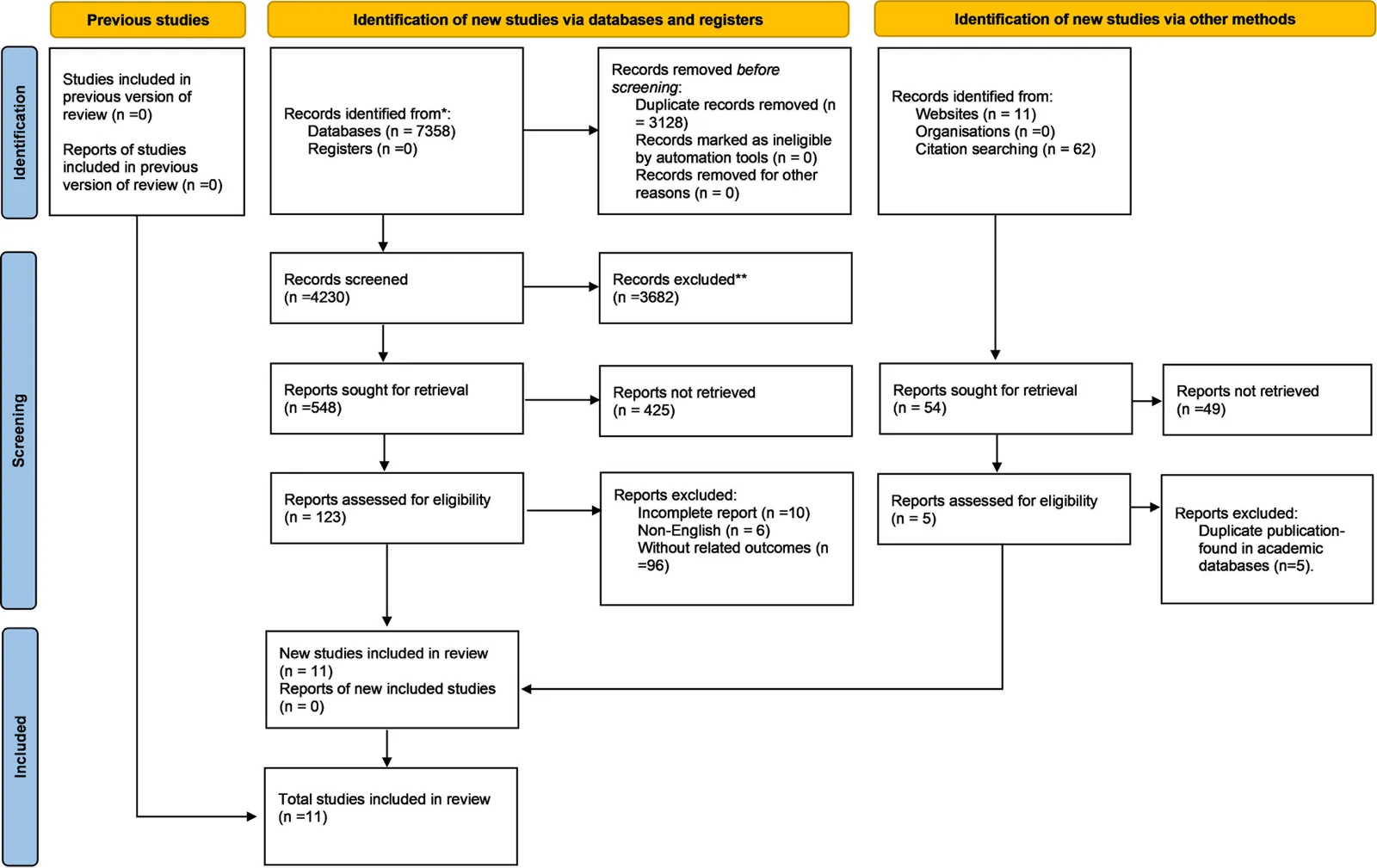
Preferred Reporting Items for Systematic Reviews and Meta-Analyses (PRISMA) 2020 flow diagram of the study identification and selection process.

The study selection process was documented at each stage. The number of studies included and excluded at both the title/abstract and full-text stages, along with the reasons for exclusion, were recorded and reported. A PRISMA flow diagram was constructed to provide a transparent visual summary of the study selection process (Fig. 1).

### 2.6. Data extraction

Data extraction was conducted independently by 2 reviewers (SYM and PP) using a standardized form to ensure consistency and minimize errors. Any discrepancies between the reviewers were discussed, and if necessary, a third reviewer was consulted to resolve disagreements. The data extracted from each study included key information related to study characteristics, population details, exposure measures, outcomes, and any risk of bias assessment.

For study characteristics, we extracted information such as the first author’s name, the year of publication, the country of origin, the study design (e.g., cohort or observational study), sample size, and the duration of follow-up. Additionally, we recorded the inclusion and exclusion criteria used in each study to assess the applicability of the findings.

Regarding the population characteristics, we collected data on patient demographics, such as age and gender, along with baseline characteristics such as eGFR stage, comorbidities (e.g., diabetes and hypertension), and baseline vitamin D levels (measured by serum 25(OH)D concentrations). We also gathered information on any specific inclusion or exclusion criteria related to the population.

For the exposure, we extracted details on how vitamin D deficiency was defined (e.g., serum 25(OH)D concentrations below 50 nmol/L or 20 ng/mL) and the methods used for assessing vitamin D levels. If vitamin D supplementation or intervention was provided, we documented the type, dose, and duration of the intervention.

The outcomes of interest included the primary outcome measures related to CKD progression, such as changes in eGFR or the rate of eGFR decline, progression to ESRD, and the need for dialysis or kidney transplantation. Secondary outcomes were also extracted when available, including changes in serum creatinine or renal fibrosis. We also gathered statistical measures, such as effect sizes, *P*-values, confidence intervals (CIs), and hazard ratios, to assess the magnitude and significance of the reported effects.

Once the data were extracted, they were cross-checked for consistency and completeness. If any information was unclear or missing, the authors of the original studies were contacted for clarification. Studies reporting multiple measures or outcomes were handled by extracting data separately for each relevant subgroup or analysis. The final data were then compiled into a database for further analysis. Throughout the process, we ensured that the data extraction was thoroughly documented, and any discrepancies were resolved through consensus between the reviewers.

In addition to the previously extracted data, for the dose-response meta-analysis, we extracted data on serum 25(OH)D levels categorized into at least 3 groups (e.g., <20 ng/mL, 20–30 ng/mL, and >30 ng/mL) or as continuous measures, including mean/median levels, number of cases, and total participants per category. If categorical data were not available, we estimated midpoints for open-ended categories based on standard methods (assuming the lowest category midpoint as 0.5 times the cutoff).

### 2.7. Quality assessment

To assess the methodological quality and risk of bias in the included studies, we used the Newcastle-Ottawa Scale (NOS), a widely recognized tool for evaluating the quality of observational studies, particularly cohort and case-control designs. The NOS tool was applied independently by 2 reviewers (SYM and PP), and any discrepancies in the quality assessment were resolved through discussion, with a third reviewer (MS) consulted if necessary.

The NOS evaluates studies based on 3 key domains:

Selection of participants: This domain assesses the process by which study participants were selected, including the representativeness of the sample, the adequacy of case definition, and whether controls were selected from the same population. Points were awarded based on the clarity and appropriateness of the selection criteria.
Comparability of cohorts: This domain evaluates how well the studies controlled for potential confounders, such as age, gender, baseline comorbidities (e.g., diabetes and hypertension), or other factors that might influence both vitamin D status and CKD progression. Studies were given points based on whether they adjusted for these confounders in their analysis.
Outcome assessment: This domain evaluates the adequacy of outcome ascertainment, follow-up length, and completeness.

Each domain in the NOS tool was rated with a set number of points, with a higher total score reflecting better study quality. The maximum possible score for cohort studies was 9 points, with studies scoring 7 or higher considered of good quality, those scoring 5 to 6 classified as fair, and studies scoring below 5 categorized as poor quality. For case-control studies, a slightly different scoring system is used, with a maximum score of 9 points.

After the individual quality assessment for each study, we synthesized the findings to provide an overall evaluation of the quality of the included studies. The results of the quality assessment were considered when interpreting the overall findings and in assessing the potential for bias across the included studies.

### 2.8. Data synthesis

The data from the included studies were synthesized using a random-effects meta-analysis approach to account for the expected heterogeneity in study designs, populations, and outcome measures. Prior to conducting the meta-analysis, we assessed the statistical heterogeneity among the studies using the I-squared heterogeneity statistic (*I*^2^) and Cochran’s *Q* test. An *I*^2^ value of 0% to 40% was considered indicative of low heterogeneity, 30% to 60% moderate heterogeneity, and above 60% high heterogeneity. Due to anticipated high heterogeneity in observational studies of this topic, we prespecified the use of a random-effects model and planned extensive exploration via subgroup analysis and meta-regression. Where heterogeneity remained extreme, we considered the relative merits of narrative synthesis versus quantitative pooling, following Cochrane recommendations. To address potential heterogeneity arising from different definitions of CKD progression, we performed prespecified and post hoc subgroup analyses stratified by outcome type (eGFR decline rate vs hard renal endpoints such as ESRD or dialysis initiation). We also performed an exploratory subgroup analysis stratified by CKD stage, categorizing studies as predominantly including patients with advanced CKD (stages 3–5) or earlier-stage CKD. Given the limited number of studies within each stratum, particularly in the earlier-stage subgroup, additional stage-specific leave-one-out sensitivity analyses were not performed.

Because the meta-analysis included a limited number of studies and substantial between-study heterogeneity was anticipated, we additionally performed a Hartung–Knapp-adjusted random-effects analysis as a sensitivity analysis. This approach uses a *t*-distribution and incorporates additional uncertainty in the estimation of the pooled effect. Results from the conventional random-effects model and the Hartung–Knapp-adjusted analysis were compared in terms of the pooled effect estimate, 95% CI, and statistical significance. The Hartung–Knapp analysis was used to assess the robustness of statistical inference and was not considered a substitute for investigating the clinical and methodological sources of heterogeneity.

For each study, we extracted the effect sizes or estimates of association (e.g., hazard ratios, odds ratios (ORs), and mean differences) along with their corresponding 95% CIs. When available, data were pooled to calculate a weighted average effect size, with weights proportional to the inverse of the variance, ensuring that larger studies contributed more to the overall estimate. We utilized STATA software version 14 (StataCorp LP) for all statistical analyses.

In cases where studies reported different types of effect sizes, we performed necessary transformations to ensure consistency across studies, such as converting correlation coefficients into pooled effect sizes or standardizing measures of CKD progression across different studies. For studies reporting continuous outcomes, we calculated the mean difference in eGFR or other relevant markers of CKD progression between participants with adequate and deficient vitamin D levels. For dichotomous outcomes, such as the development of ESRD or the need for dialysis, we used ORs or risk ratios, as appropriate.

If significant heterogeneity was detected in the meta-analysis, subgroup analyses were planned to explore potential sources of variability, including differences in study design (e.g., cohort vs observational), follow-up duration, study quality score, regional differences, CKD stages, and the presence of coexisting conditions such as diabetes or hypertension. Given the limited number of included studies, meta-regression analyses were conducted as exploratory univariable models rather than as a multivariable model. Study-level characteristics, including sample size, follow-up duration, and study quality, were evaluated separately as potential moderators of the pooled association. For each meta-regression model, we reported the residual between-study variance (τ^2^), residual *I*^2^, and the proportion of between-study variance explained by the moderator (*R*^2^ analog), calculated from the reduction in τ^2^ relative to the unadjusted random-effects model. Because only 11 studies were available, the meta-regression findings were considered exploratory and hypothesis-generating. A leave-one-out sensitivity analysis was performed to evaluate the influence of each study on the overall effect size.

Potential small-study effects and publication bias were assessed using visual inspection of the funnel plot and Begg’s and Egger’s tests. Because these statistical tests have limited power when the number of included studies is small, a nonsignificant result was not considered evidence of the absence of publication bias.

Because the included studies used heterogeneous definitions of CKD progression, the overall meta-analysis was considered a broad synthesis across related but clinically nonequivalent renal progression outcomes. To improve clinical interpretability, outcome-specific analyses were conducted at the broadest level supported by the available data, distinguishing kidney-function decline from hard or advanced renal progression endpoints. Further disaggregation into individual outcomes such as ESRD, dialysis initiation, and study-defined composite outcomes was not quantitatively undertaken when only a small number of independent studies were available. These narrowly defined outcomes were instead summarized descriptively at the study level.

### 2.9. Dose-response meta-analysis

To further characterize the association between serum 25(OH)D concentrations and CKD progression, we conducted a dose-response meta-analysis using the 2-stage generalized least-squares trend estimation method proposed by Greenland and Longnecker, as implemented in STATA version 14. This approach was used to evaluate both linear and potential nonlinear associations.

For studies reporting serum 25(OH)D concentrations in categories, the category-specific mean or median concentration was used as the representative exposure value whenever available. When these values were not reported, the midpoint between the lower and upper boundaries of each category was assigned. For an open-ended lowest category, the representative value was estimated as one-half of the upper boundary, whereas for an open-ended highest category, the representative value was estimated as 1.5 times the lower boundary. All serum 25(OH)D concentrations were standardized to ng/mL before analysis. Studies reporting fewer than 3 exposure categories were included only when a directly reported continuous effect estimate was available; otherwise, they were excluded from the dose-response modeling.

The linear association was estimated for each 10 ng/mL decrement in serum 25(OH)D concentration. Potential nonlinearity was assessed using restricted cubic splines with knots placed at the 10th, 50th, and 90th percentiles of the exposure distribution. Between-study heterogeneity was evaluated using the *I*^2^ statistic. Exploratory meta-regression analyses were also performed to examine whether study-level characteristics, including CKD stage and follow-up duration, were associated with variation in the dose-response estimates.

Because the dose-response analysis was based on aggregate data from a limited number of studies and required the estimation of representative exposure values for some categories, the resulting curve was interpreted as an approximate study-level representation of the association. These assumptions may have introduced exposure misclassification and limited the precision with which the exact shape of the association, particularly nonlinearity, could be determined.

### 2.10. GRADE assessment

The quality of evidence for the primary outcomes (CKD progression, eGFR decline, and ESRD) was evaluated using the Grading of Recommendations Assessment, Development and Evaluation (GRADE) approach. GRADE rates evidence as high, moderate, low, or very low based on 5 domains: risk of bias (informed by NOS scores), inconsistency (heterogeneity via *I*^2^), indirectness (applicability to the CKD population), imprecision (width of CIs), and publication bias (Egger’s test). Two reviewers (SYM and PP) independently applied GRADE, with discrepancies resolved by consensus. Upgrades were considered for large effect sizes or dose-response gradients. Results are presented in a GRADE evidence profile table.

## 3. Results

### 3.1. Characteristics of included studies

As shown in Figure 1, 7358 records were obtained from the systematic search process. After removing duplicate records (n = 3128), 4230 articles were included in the first stage of screening. In the second stage of screening, the full texts of 123 articles were evaluated, and finally, 11 studies were included in the final analysis.^[19–30]^ Among the included studies, 4 were conducted in China,^[19,25,26,29]^ 2 in Italy,^[21,22]^ and others were conducted in Thailand,^[23]^ Japan,^[28]^ Georgia,^[24]^ Australia,^[20]^ and Korea.^[27]^ The sample size of the included studies varied between 182 and 36,523 participants. All the included studies were conducted between 2013 and 2024. Table 1 summarizes the baseline characteristics of the included studies. In terms of the quality assessment, all the included studies were of higher quality (score >5; Table 2).

**Table 1:** Characteristics of included studies.

Study	Inclusion criteria (definition of CKD)	Study design	Sample size	Follow-up time	Men	Mean age	BMI	Mean vitamin D level	Mean eGFR	Outcomes	Quality score
Mai et al^[19]^	eGFR, <60 mL/min per 1.73 m^2^ and/or an elevated urinary albumin-creatinine ratio (ACR, ≥30 mg/g)	Cohort	4989	70 mo	2342	63.45 ± 7.62	NR	NR	48.34 ± 5.26	Mortality outcomes, CKD progression	6
Herrmann et al^[30]^	Patient with deteriorating renal function, Scr >130 mol/L	Cohort	4964	5 yr	2732	62 ± 7	30.4 ± 8.36	49 nmol/L	NR	Microvascular complications	7
Galassi et al^[21]^	20% reduction in eGFR or dialysis initiation at 6 mo	Cohort	71	6 mo	54	75 (69–80)	27.3 (24.5–32.2)	31.0 (22.7–41.8)	31.2	Albuminuria, CKD progression	5
Duan et al^[22]^	Predialytic CRF patients, Ccr 20–50 mL/min	Cohort	182	42 mo	137	52 ± 11	25 (23, 27)	26 (22, 30)	51 (28, 73)	CKD progression	7
Xie et al^[29]^	UACR > 300 mg/g	Cross-sectional	351	5 yr	242	56.0 ± 9.9	26.1 ± 3.6	17.30 ± 6.30	NR	Diabetic kidney disease	6
Feng et al^[25]^	eGFR < 60	Cross-sectional	152	2 yr	78	59.3 ± 15.7	NR	27.2 ± 6.2	21.1 ± 14.9	eGFR decline	6
Guessous et al^[24]^	eGFR < 60	Cohort	4280	5.5 yr	1960	52.5 ± 10.5	NR	24.5 ± 2.8	46.2 ± 14.8	Rapid eGFR decline	7
Satirapoj et al^[23]^	eGFR < 60	Cross-sectional	2895	2 yr	1516	63.34 ± 16.89	NR	24.09 ± 11.65	33.22 ± 17.99	ESRD occurrence	8
Izumaru et al^[28]^	eGFR < 60	Cohort	2417	5 yr	1195	60 ± 11	23.2 ± 3.4	38.15 ± 9.23	NR	eGFR decline	6
Jhee et al^[27]^	Ccr 15–70 mL/min, stable for 3 mo	Cross-sectional	2693	2 yr	1324	48.1 ± 15.9	23.6 ± 3.3	17.9 ± 6.5	NR	Renal hyperfiltration	7
Liu et al^[26]^	ACR > 30 mg/g and eGFR < 60	Cohort	1037	4 yr	507	85.44 ± 11.66	21.36 ± 3.90	37.63 ± 16.40	53.76 ± 9.67	Albuminuria and impaired GFR incidence	8

ACR = albumin-creatinine ratio, CKD = chronic kidney disease, eGFR = estimated glomerular filtration rate, ESRD = end-stage renal disease, GFR = glomerular filtration rate, NR = not reported, Scr = serum creatinine, UACR = urine albumin-creatinine ratio.

**Table 2:** The quality of cohort studies using the Newcastle-Ottawa Scale.

Author	Representativeness of the exposed cohort	Selection of the nonexposed cohort	Ascertainment of exposure	Outcome of interest was not present at start of study	Adjustment for main confounding factor	Controls for any additional factor	Assessment of outcome	Follow-up long enough	Adequacy of follow-up of cohorts	Total
Mai et al^[19]^	*			*	*		*	*	*	6
Herrmann et al^[30]^	*		*		*	*	*	*	*	7
Galassi et al^[21]^	*	*		*			*	*		5
Duan et al^[22]^	*	*		*	*		*	*	*	6
Xie et al^[29]^	*	*	*	*		*			*	6
Feng et al^[25]^		*	*	*			*	*	*	6
Guessous et al^[24]^	*	*		*	*	*	*		*	7
Satirapoj et al^[23]^	*		*	*	*	*	*	*	*	8
Izumaru et al^[28]^		*		*		*	*	*	*	6
Jhee et al^[27]^	*			*	*	*	*	*	*	7
Liu et al^[26]^	*	*	*	*	*		*	*	*	7

### 3.2. Association between vitamin D deficiency and CKD progression

**Figure 2.**
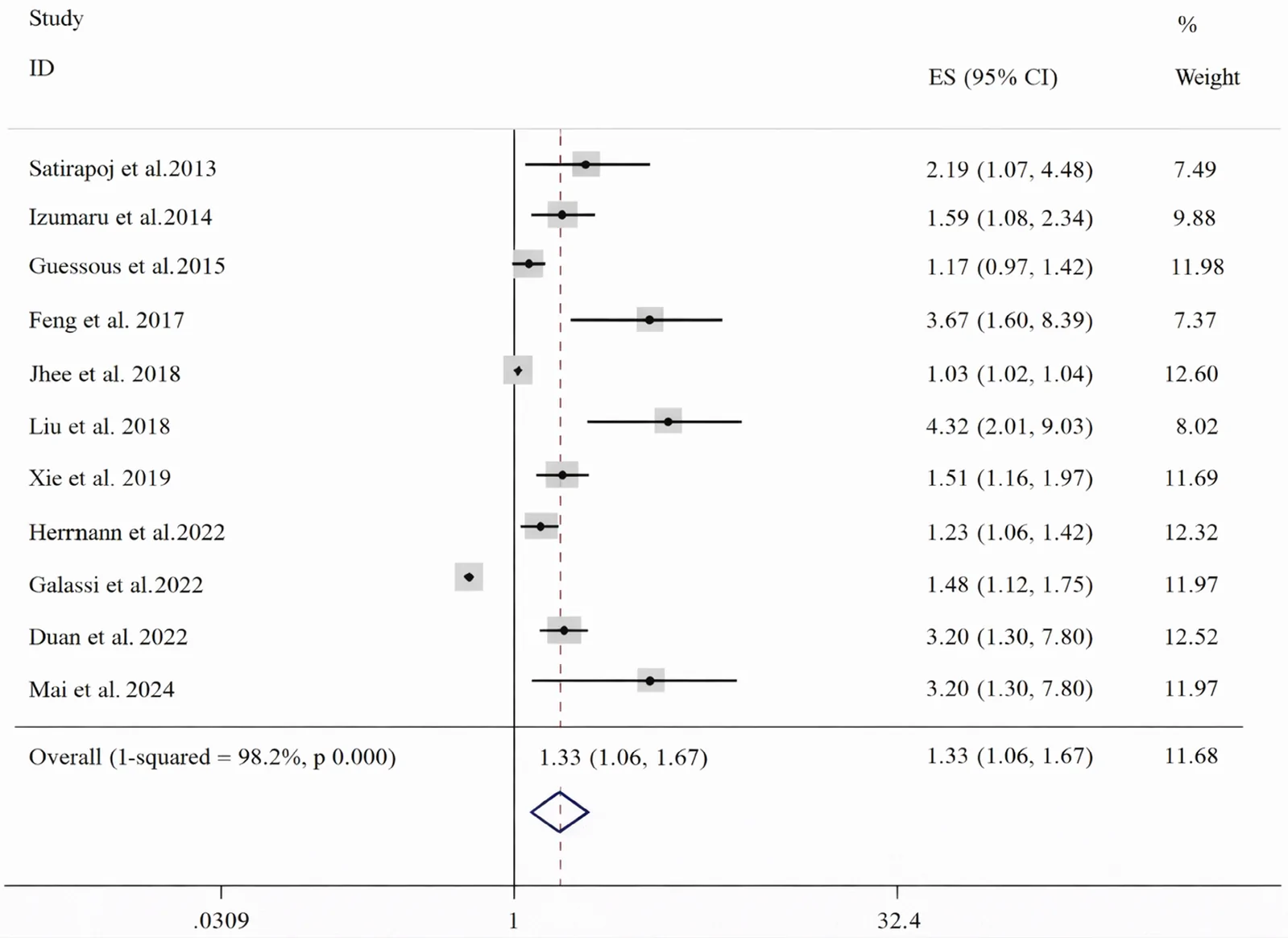
Forest plot of odds ratios and 95% confidence intervals for the association between vitamin D deficiency and chronic kidney disease progression. CI = confidence interval, ER = effect size, lnHR = natural logarithm of the hazard ratio.

Eleven studies evaluated the association between vitamin D deficiency and CKD progression. The pooled analysis showed that vitamin D deficiency was associated with higher odds of CKD progression compared with adequate vitamin D status (OR = 1.33, 95% CI: 1.06–1.67; *P* = .019). However, between-study heterogeneity was considerable (*I*^2^ = 98.2%; *P* < .001; Fig. 2). The Hartung–Knapp-adjusted sensitivity analysis yielded a pooled OR of 1.33 (95% CI: 1.03–1.72; *P* = .034). Although the point estimate was essentially unchanged from that obtained using the conventional random-effects model (OR = 1.33, 95% CI: 1.06–1.67), the wider CI reflected greater uncertainty after accounting for the limited number of studies. Nevertheless, the direction and statistical significance of the association were retained.

Accordingly, the pooled estimate should be interpreted as an average association across heterogeneous study populations, CKD stages, follow-up durations, exposure definitions, and progression outcomes, rather than as a single universally applicable effect size. Although the study-specific estimates were generally in the same direction, their magnitudes varied substantially.

In the subgroup analysis, we found a significant association between vitamin D deficiency and CKD progression only in studies with long-term follow-up (≥5 years; OR = 2.07 [95% CI: 1.29–3.14]; *P* = .001), cohort studies (OR = 1.87 [95% CI: 1.19–3.18]; *P* = .003), high-quality studies (quality score ≥7; OR = 1.35 [95% CI: 1.06–1.69]; *P* = .03), and studies including patients with other comorbidities (OR = 1.38 [95% CI: 1.19–1.76]; *P* < .001; Table 3).

**Table 3:** Subgroup analyses for the association between vitamin D deficiency and CKD progression.

	Number of effect sizes	OR (95% CI)	*P* effect	*P* heterogeneity	*I*^2^ (%)	*P* between
Follow-up						.001
<5 yr	5	1.08 (0.59–1.78)	.69	<.001	98.1	
≥5 yr	6	2.07 (1.29–3.14)	.001	<.001	89.3	
Design						<.001
Cohort	7	1.87 (1.19–3.18)	.003	<.001	86.5	
Cross-sectional	4	1.05 (0.86–1.12)	.39	<.001	97.1	
Study quality score						.001
<7	6	1.29 (0.71–2.39)	.39	<.001	95.9	
≥7	5	1.35 (1.06–1.69)	.03	<.001	82.7	
Presence of comorbidity						<.001
Yes	6	1.38 (1.19–1.76)	<.001	<.001	77.9	
No	5	1.27 (0.78–2.10)	.32	<.001	96.3	
Regional differences						.01
Asia	8	1.65 (1.24–2.19)	<.001	.16	49.3	
Europe and Australia	3	1.28 (0.92–1.78)	.38	<.001	98.33	
Outcome type						.03
eGFR decline	6	1.42 (1.15–1.76)	<.001	.23	47.36	
ESRD/hard endpoints	5	1.51 (1.12–2.03)	<.001	<.001	97.84	
CKD stages						.019
CKD 3–5	7	1.83 (1.26–2.32)	<.001	.078	44.33	
Earlier-stage	4	1.16 (0.85–1.55)	.28	<.001	96.37	

CI = confidence interval, CKD = chronic kidney disease, eGFR = estimated glomerular filtration rate, ESRD = end-stage renal disease, WMD = weighted mean difference.

Subgroup analyses suggested that geographic region and CKD stage might have contributed to the observed heterogeneity. However, because the analyses were based on a limited number of studies within each subgroup, these findings should be interpreted as exploratory. Studies conducted in Asia exhibited stronger associations between vitamin D deficiency and CKD progression (OR = 1.65, 95% CI: 1.24–2.19) compared with studies from Europe and Australia (OR = 1.28, 95% CI: 0.92–1.78), highlighting potential geographical variations. Moreover, stage-stratified subgroup analysis showed a stronger association among studies including patients with advanced CKD (stages 3–5; OR = 1.83, 95% CI: 1.26–2.32) than among studies of earlier-stage CKD (OR = 1.16, 95% CI: 0.85–1.55), with a statistically significant between-subgroup difference (*P* = .019). However, because only 7 and 4 effect estimates contributed to the advanced- and earlier-stage subgroups, respectively, these findings should be considered exploratory.

Because the definitions of CKD progression varied substantially across studies, the overall pooled estimate should be regarded as a broad summary association rather than as an effect estimate for a single clinically homogeneous endpoint. To provide greater clinical specificity, we conducted separate analyses for the 2 outcome categories supported by an adequate number of effect estimates. Among studies evaluating eGFR decline, vitamin D deficiency was associated with higher odds of deterioration in kidney function (OR = 1.42, 95% CI: 1.15–1.76). Among studies evaluating hard or advanced renal progression outcomes, including ESRD and dialysis-related endpoints, the pooled OR was 1.51 (95% CI: 1.12–2.03). However, substantial heterogeneity remained in the latter group. Further quantitative separation of ESRD, dialysis initiation, and individual composite outcomes was not performed because each narrowly defined endpoint was represented by only a small number of studies. These outcomes are reported separately at the study level in Table 1 and were not subjected to potentially unstable outcome-specific meta-analyses.

Exploratory meta-regression indicated that studies with longer follow-up (≥5 years) reported larger associations between vitamin D deficiency and CKD progression (*P* = .002). This study-level finding should not be interpreted as evidence of a cumulative causal effect.

**Figure 3.**
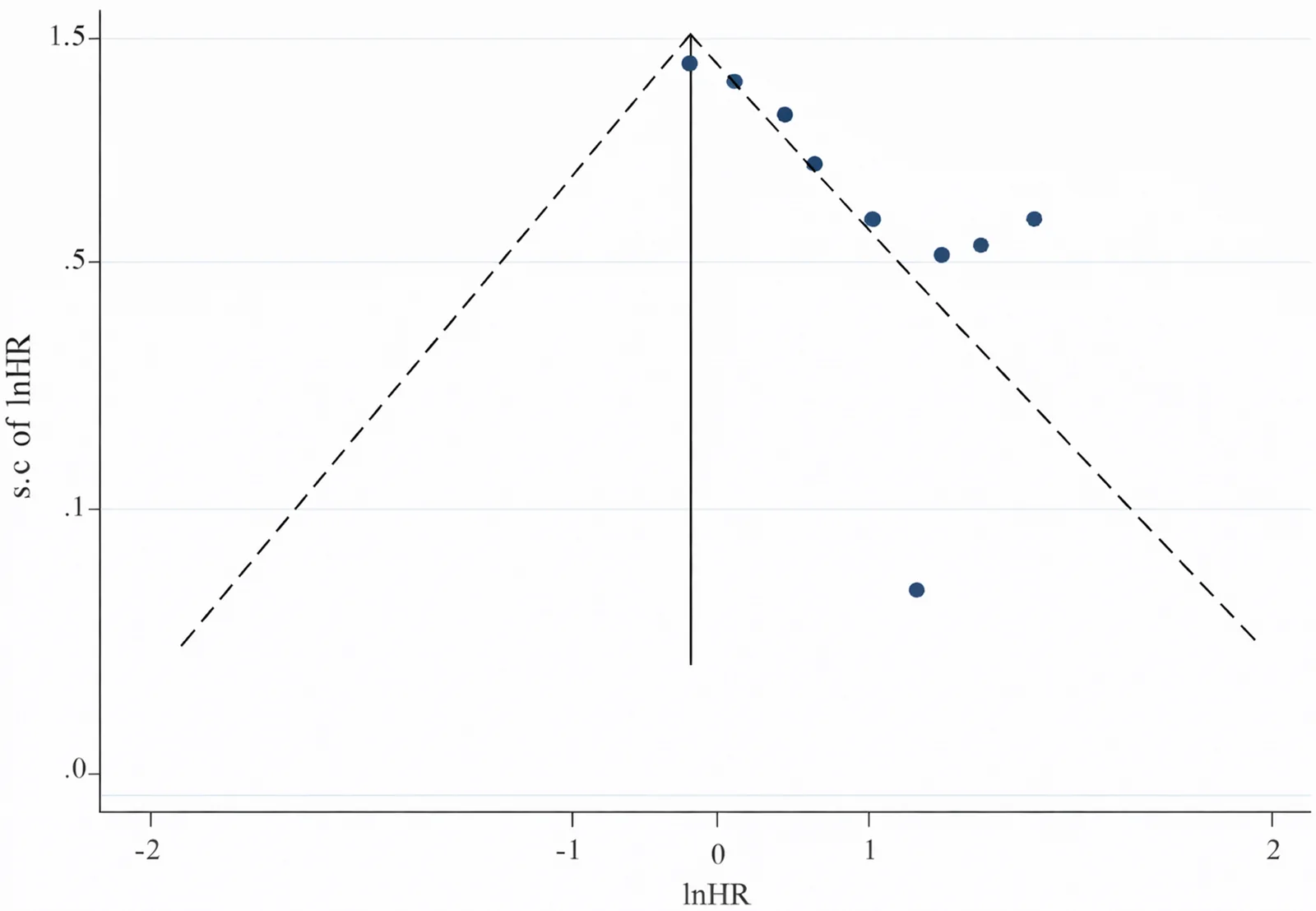
Funnel plots detailing publication bias in the studies that evaluated the association between vitamin D deficiency and CKD progression. CKD = chronic kidney disease.

The leave-one-out sensitivity analysis showed that omitting any single study did not materially alter the pooled effect size. Visual inspection of the funnel plot suggested asymmetry (Fig. 3). Begg’s test (*P* = .33) and Egger’s regression test (*P* = .14) were not statistically significant. However, because only 11 studies were included and these tests have limited statistical power in small meta-analyses, publication bias could not be excluded. The trim-and-fill analysis did not materially change the direction of the pooled association; nevertheless, this exploratory analysis cannot rule out publication bias or other small-study effects.

### 3.3. Dose-response meta-analysis

Dose-response data were available from 10 studies. The analysis suggested that each 10 ng/mL lower serum 25(OH)D concentration was associated with higher odds of CKD progression (OR = 1.18, 95% CI: 1.09–1.25; *P* < .001), with substantial heterogeneity (*I*^2^ = 78.5%; *P* = .002). There was no statistically significant evidence of departure from linearity (*P* for nonlinearity = .19). However, given the limited number of contributing studies and the use of estimated representative doses for some exposure categories, the analysis had limited ability to characterize the precise shape of the association. The curve should therefore be interpreted as suggesting an approximately linear association within the range of the available data rather than establishing a strictly linear relationship.

**Figure 4.**
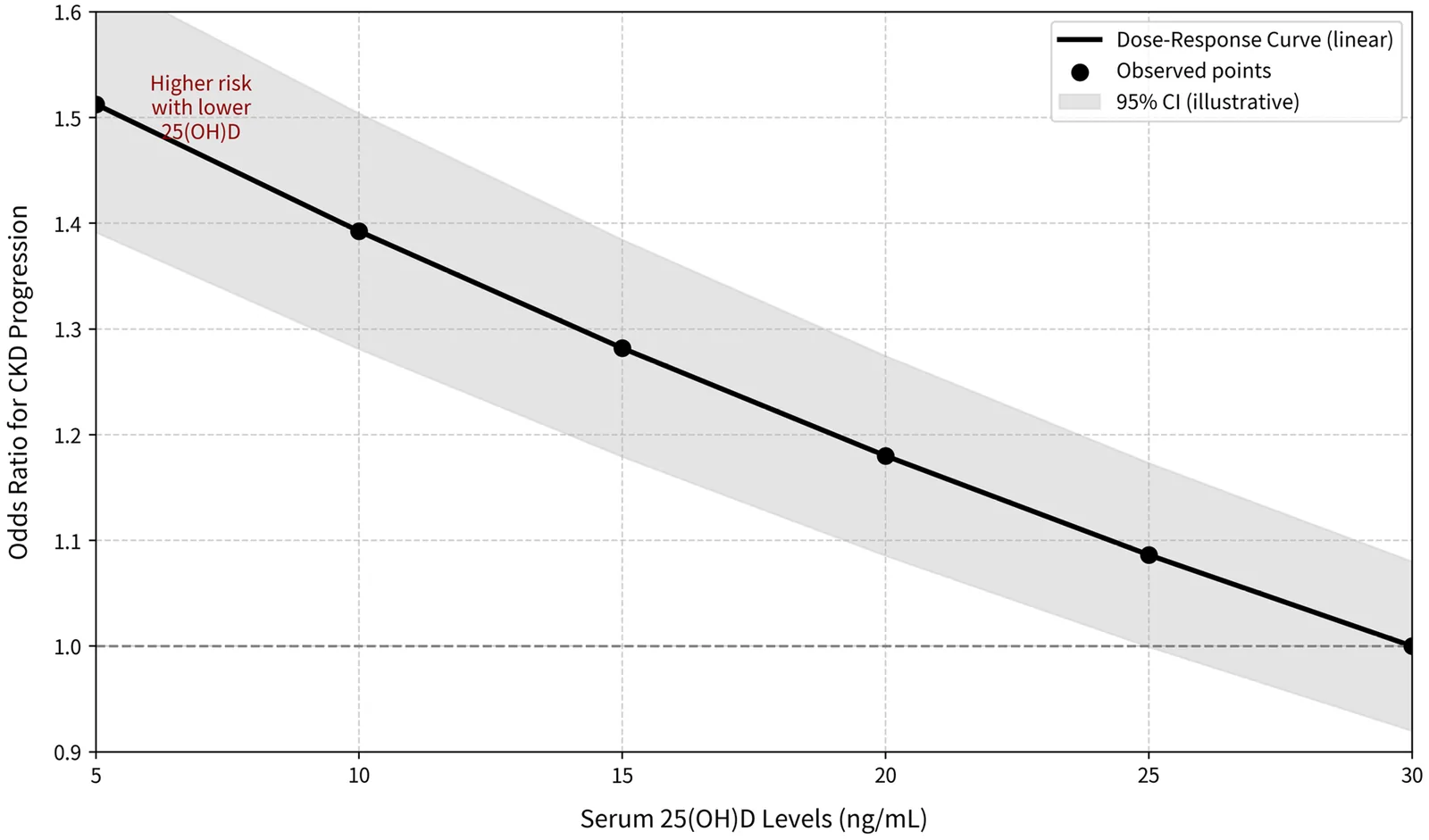
Dose-response curve for serum 25-hydroxyvitamin D (25(OH)D) and chronic kidney disease progression risk, using 30 ng/mL as the reference concentration. The curve suggests an approximately linear increase in risk as 25(OH)D concentrations decrease. CI = confidence interval, CKD = chronic kidney disease.

Exploratory univariable meta-regression indicated that follow-up duration was associated with variation in the reported effect estimates (*P* = .002). After inclusion of follow-up duration as a moderator, substantial residual heterogeneity remained (residual τ^2^ = 0.08, residual *I*^2^ = 92.5%), with follow-up duration explaining approximately 35% of the estimated between-study variance. Thus, although follow-up duration accounted for a proportion of the observed heterogeneity, considerable unexplained heterogeneity remained. Figure 4 presents the dose-response curve (reference at 30 ng/mL), illustrating the linear increase in risk with decreasing 25(OH)D levels.

### 3.4. GRADE assessment

The certainty of evidence was reassessed using the GRADE framework (Table 4). For the overall association between vitamin D deficiency and CKD progression, the evidence was rated as very low certainty. The body of evidence initially started at low certainty because all included studies were observational and was downgraded by 1 level for serious inconsistency, given the considerable and largely unexplained between-study heterogeneity (*I*^2^ = 98.2%). Although subgroup analyses and exploratory meta-regression identified study-level characteristics that may have contributed to the observed variability, substantial residual heterogeneity remained. The possibility of publication bias and residual confounding further reduced confidence in the pooled estimate, although no additional formal downgrading was applied because the certainty had already reached the very-low category.

**Table 4:** GRADE evidence profile for the association between vitamin D deficiency and CKD progression.

Outcome	No. of contributing studies/estimates	Risk of bias	Inconsistency	Indirectness	Imprecision	Publication bias	Other considerations	Certainty of evidence (GRADE)
CKD progression (overall pooled association)	11	Not serious overall; residual and unmeasured confounding remain possible because all evidence is observational	Serious (*I*^2^ = 98.2%; considerable and largely unexplained heterogeneity persisted after subgroup analyses and meta-regression)	Serious (the pooled endpoint combines clinically nonequivalent outcomes, including eGFR decline, ESRD, dialysis-related events, and composite renal worsening)	Not serious	Could not be excluded (funnel-plot asymmetry and limited power of statistical tests with only 11 studies)	None	Very low
Dose-response association (per 10 ng/mL decrement in 25(OH)D)	10	Not serious overall; observational design and residual confounding remain important limitations	Serious (*I*^2^ = 78.5%)	Not serious	Not serious	Could not be reliably excluded because of the limited number of contributing studies	Observed dose-response gradient (upgrade by 1 level), interpreted cautiously because some exposure values were reconstructed from category midpoints/open-ended intervals	Low
eGFR decline	6	Not serious overall; residual confounding remains possible	Not serious to moderate (*I*^2^ = 47.4%); no formal downgrade for inconsistency	Not serious	Not serious	Could not be reliably assessed because fewer than 10 studies contributed	None	Low
Hard or advanced renal progression outcomes (including ESRD and dialysis-related endpoints)	5	Not serious overall; residual confounding remains possible	Serious (*I*^2^ = 97.8%; substantial unexplained heterogeneity)	Not serious	Not serious	Could not be reliably assessed because of the small number of studies	None	Very low

Under the conventional GRADE framework used in this review, certainty started at low because the evidence base consisted entirely of observational studies.

For the overall CKD progression outcome, certainty was downgraded for serious inconsistency (*I*^2^ = 98.2%). The broad pooled endpoint also combined clinically nonequivalent outcome definitions; therefore, the overall estimate should be interpreted only as a broad summary association. The final certainty was rated very low.

For the dose-response analysis, certainty was downgraded for serious inconsistency (*I*^2^ = 78.5%) and upgraded by 1 level for the observed dose-response gradient, resulting in low-certainty evidence. This upgrade should be interpreted cautiously because the analysis included only 10 studies and some representative exposure values were reconstructed using category midpoints and assumptions for open-ended intervals.

For eGFR decline, the updated outcome-specific analysis included 6 contributing estimates and showed moderate heterogeneity (*I*^2^ = 47.4%); no formal downgrade for inconsistency was applied, resulting in low-certainty evidence.

For hard or advanced renal progression outcomes, 5 contributing estimates were available, and heterogeneity remained very high (*I*^2^ = 97.8%); the evidence was therefore downgraded for serious inconsistency to very low certainty.

Publication bias could not be excluded. The overall funnel plot showed asymmetry, while Begg’s and Egger’s tests had limited power because of the small number of included studies. For outcome-specific analyses with fewer than 10 studies, formal assessment of publication bias was considered unreliable.

25(OH)D = 25-hydroxyvitamin D, CKD = chronic kidney disease, eGFR = estimated glomerular filtration rate, ESRD = end-stage renal disease, GRADE = Grading of Recommendations Assessment, Development and Evaluation, NOS = Newcastle-Ottawa Scale.

For the dose-response analysis, the certainty of evidence was rated as low. The evidence started at low certainty because of the observational study designs and was downgraded for serious inconsistency (*I*^2^ = 78.5%). The observed dose-response gradient was considered a factor supporting an upgrade by 1 level; however, confidence in this finding was tempered by the limited number of contributing studies and the need to estimate representative 25(OH)D exposure values for some categories. Accordingly, the dose-response findings should be interpreted as supportive of an association rather than as evidence of a precise causal or therapeutic relationship.

## 4. Discussion

This systematic review and meta-analysis aimed to examine the association between serum vitamin D levels (specifically 25(OH)D) and the progression of CKD. The findings showed that lower serum 25(OH)D levels were associated with higher odds of CKD progression. This association was observed across several analyses; however, the observational nature of the evidence precludes conclusions regarding causality. The direction of the pooled association remained unchanged in the Hartung–Knapp-adjusted sensitivity analysis. However, the wider CI emphasized the uncertainty arising from the small number of studies and the considerable between-study heterogeneity. Thus, concordance in the direction of the estimates should not be interpreted as eliminating concerns regarding heterogeneity or generalizability.

An important interpretive consideration is that the included studies did not use a uniform definition of CKD progression. A decline in eGFR reflects worsening kidney function, whereas ESRD and dialysis initiation represent more advanced and clinically consequential outcomes. Composite renal endpoints may additionally combine events with substantially different clinical importance and incidence. Accordingly, the overall pooled estimate should not be interpreted as representing a single uniform clinical outcome. The outcome-specific analyses provide greater clinical context, although the limited number of studies prevented reliable quantitative pooling of individual endpoints. Future studies should adopt standardized and hierarchically defined renal outcomes to improve comparability across cohorts and meta-analyses.

Our findings align with several prior studies that have suggested an association between low vitamin D levels and adverse renal outcomes. Wu et al, in a meta-analysis of 9 observational studies, found that adequate 25(OH)D levels were associated with a lower risk of renal cell carcinoma.^[31]^ Similarly, Derakhshanian et al reported a significantly higher risk of diabetic nephropathy among patients with diabetes and vitamin D deficiency.^[32]^ Our subgroup analysis, which found a significant association between vitamin D deficiency and CKD progression in studies with long-term follow-up (≥5 years) and high-quality designs, supports the notion that longer durations of observation and methodological rigor may be crucial in detecting the effects of vitamin D on renal function.

The dose-response analysis suggested that lower serum 25(OH)D concentrations were associated with progressively higher odds of CKD progression, with an estimated OR of 1.18 per 10 ng/mL decrement. Although the test for nonlinearity was not statistically significant, this should not be interpreted as definitive evidence of a strictly linear relationship. The limited number of contributing studies, substantial heterogeneity, and reliance on reconstructed category-level exposure values reduced the precision with which the shape of the association could be characterized.

The considerable between-study heterogeneity (*I*^2^ = 98.2%) represents the principal limitation of the quantitative synthesis. Although subgroup analyses and meta-regression suggested that follow-up duration, geographic region, CKD stage, and comorbidity status might contribute to the observed variability, the small number of included studies limited the reliability of these analyses, and substantial residual heterogeneity remained. Consequently, the pooled OR should be understood as an average association across heterogeneous clinical and methodological settings rather than as a universal estimate applicable to all CKD populations. Although the direction of most study-specific estimates was broadly consistent, the magnitude of the association varied considerably. The subgroup and meta-regression findings should, therefore, be regarded as exploratory and hypothesis-generating.

Given the considerable heterogeneity, we acknowledge that a single pooled OR should be interpreted with caution and may not fully represent a universal effect size. In accordance with Cochrane Handbook guidance,^[33]^ when heterogeneity remains largely unexplained after exploration, a narrative synthesis may be more appropriate than relying solely on a pooled estimate, particularly for clinical decision-making in heterogeneous populations. However, because the direction of association was consistent across 10 of 11 studies, the dose-response meta-analysis showed a clear linear gradient (*P* for nonlinearity = .19), and sensitivity analyses confirmed stability of the overall finding, we retained the random-effects pooled estimate as the primary summary while emphasizing its limitations. We present subgroup-specific estimates as more clinically interpretable in defined contexts. The very-low certainty rating for the overall association and low certainty rating for the dose-response analysis reflect these limitations, despite the supportive dose-response gradient.

The stronger association observed in studies with longer follow-up may reflect differences in cumulative exposure, disease severity, outcome ascertainment, or residual confounding. Because this finding was derived from study-level subgroup and meta-regression analyses, it should not be interpreted as evidence that prolonged vitamin D deficiency causally accelerates renal function decline. The leave-one-out analysis showed that the direction of the pooled association was not dependent on any single study; however, this finding does not eliminate residual confounding or establish causality.

The kidney is a primary target organ for vitamin D, as evidenced by the high expression of the vitamin D receptor within its tissues. This observation aligns with findings from different studies that demonstrated an inverse relationship between serum 25(OH)D concentration and the prevalence of albuminuria.^[34,35]^ The association between vitamin D deficiency and CKD progression is underpinned by several biological mechanisms that influence kidney function, inflammation, and mineral metabolism. Vitamin D, primarily in its active form (1,25-dihydroxyvitamin D), exerts a wide range of effects through its binding to the vitamin D receptor in various tissues, including the kidney. These effects are crucial for maintaining homeostasis in calcium and phosphate metabolism, modulating immune responses, and preventing fibrosis and vascular damage. Specifically, vitamin D exhibits renoprotective properties by suppressing the renin–angiotensin–aldosterone system (RAAS), which reduces intraglomerular pressure and proteinuria – a key marker of CKD progression.^[36]^ Additionally, it inhibits transforming growth factor-beta signaling pathways, thereby attenuating renal fibrosis through decreased extracellular matrix deposition and fibroblast activation.^[37–39]^ Several biological pathways may provide plausibility for the observed association between lower vitamin D status and CKD progression. These proposed mechanisms include alterations in RAAS activity, inflammatory signaling, mineral metabolism, and renal fibrosis. However, the present meta-analysis cannot determine whether these pathways mediate a causal effect of vitamin D deficiency on kidney function.^[40–42]^ These mechanistic considerations should be viewed as supportive biological context rather than evidence that the observed epidemiological association is causal.

Vitamin D signaling has been linked to regulation of the RAAS, mineral metabolism, inflammatory responses, and pathways involved in renal fibrosis. Disturbances in vitamin D metabolism are also common as kidney function declines and may coexist with secondary hyperparathyroidism, altered phosphate homeostasis, and systemic inflammation. These mechanisms provide biological plausibility for an association between vitamin D status and renal outcomes; however, they do not establish that vitamin D deficiency is a direct cause of CKD progression. Importantly, the relationship may also operate in the opposite direction because declining kidney function itself can alter vitamin D metabolism and circulating 25(OH)D concentrations. Therefore, the mechanistic evidence should be considered supportive biological context rather than proof of a causal pathway or therapeutic benefit from vitamin D supplementation.^[36,43–47]^

This meta-analysis provides an updated synthesis of the association between vitamin D status and CKD progression and incorporates subgroup, dose-response, and sensitivity analyses to examine the consistency and potential modifiers of the observed association. The inclusion of clinically relevant renal outcomes and the assessment of evidence certainty using GRADE provide additional context for interpretation. Nevertheless, these methodological strengths should be weighed against the considerable heterogeneity, the limited number of available studies, heterogeneous outcome definitions, and the observational nature of the evidence. Accordingly, the findings are most appropriately interpreted as identifying a potentially relevant prognostic association that warrants further investigation rather than as evidence supporting a causal relationship or a specific clinical intervention.

Despite the strengths of this meta-analysis, several limitations should be considered when interpreting the findings. First, all included studies were observational; therefore, the pooled estimates represent associations and should not be interpreted as evidence of causality. Although several primary studies adjusted their estimates for measured confounders, residual and unmeasured confounding cannot be excluded. Reverse causation is also plausible because deterioration in kidney function may itself alter vitamin D metabolism and circulating 25(OH)D concentrations. Accordingly, the observed association does not establish that vitamin D deficiency independently causes CKD progression or that correction of vitamin D deficiency would reduce the risk of renal function decline.

Second, the considerable between-study heterogeneity (*I*^2^ = 98.2%) represents a major limitation of the quantitative synthesis. Although subgroup analyses and meta-regression were performed to explore potential sources of heterogeneity, substantial residual variability remained. Differences in study populations, CKD stage, follow-up duration, comorbidities, vitamin D assessment methods, and definitions of renal progression may all have contributed to the observed heterogeneity. In addition, only 11 studies were included, limiting the reliability and statistical power of subgroup analyses and meta-regression. The pooled effect should therefore be interpreted as an average association across clinically and methodologically heterogeneous studies rather than as a universally applicable effect estimate.

The limited number of included studies also affected the assessment of statistical uncertainty and potential small-study effects. Although a Hartung–Knapp-adjusted sensitivity analysis was performed to account more fully for uncertainty associated with a small evidence base, no statistical adjustment can overcome the underlying limitations imposed by sparse data and substantial clinical and methodological heterogeneity. Moreover, inference based on the Hartung–Knapp approach may itself remain uncertain when few studies with markedly different precisions contribute to the analysis. Consequently, both the conventional and Hartung–Knapp-adjusted estimates should be interpreted cautiously. Similarly, although Begg’s and Egger’s tests were not statistically significant, the limited power of these tests in a meta-analysis of only 11 studies, together with the observed funnel-plot asymmetry, means that publication bias and other small-study effects cannot be excluded.

Third, the definition of CKD progression was not uniform across the included studies and encompassed eGFR decline, ESRD, dialysis-related outcomes, and study-defined composite renal endpoints. Although outcome-specific analyses were conducted at the broadest clinically meaningful level supported by the available data, further disaggregation into individual endpoints was limited by the small number of studies contributing to each narrowly defined outcome. As a result, some outcome-specific effects could not be estimated with sufficient precision and should be interpreted cautiously. Furthermore, studies in which proteinuria or albuminuria constituted the primary renal outcome were excluded because the present review focused primarily on eGFR decline and progression to ESRD. Although this approach improved consistency with the prespecified definition of CKD progression, it may have reduced the comprehensiveness of the evidence base by excluding clinically relevant markers of kidney damage and progression.

The dose-response findings should also be interpreted with caution. The dose-response analysis was based on only 10 studies, which limited the precision of the estimated curve and the statistical power to distinguish linear from nonlinear patterns. In several studies, serum 25(OH)D concentrations were reported in categories without corresponding category-specific means or medians; therefore, representative exposure values had to be reconstructed using category midpoints and assumptions for open-ended intervals. These procedures assign a single representative exposure value to all participants within a category and may not accurately reflect the underlying distribution of vitamin D concentrations, particularly for broad or open-ended categories. Differences in vitamin D assays, exposure thresholds, and baseline 25(OH)D ranges across studies may have introduced additional measurement heterogeneity. Therefore, the observed dose-response pattern should be regarded as an approximate study-level association rather than evidence of a precise biological relationship or a clinically actionable therapeutic threshold.

Taken together, these limitations reduce confidence in the precision and generalizability of the pooled estimates. Future research should include larger, well-designed prospective studies using standardized definitions of CKD progression and more consistent assessment of vitamin D status. Studies incorporating both eGFR-based outcomes and proteinuria or albuminuria may provide a more comprehensive evaluation of renal progression. Ultimately, adequately powered randomized controlled trials are required to determine whether correction of vitamin D deficiency can improve clinically relevant kidney outcomes.

### 4.1. Clinical interpretation and implications

The present findings indicate that lower serum 25(OH)D concentrations may be associated with a greater likelihood of CKD progression, but their immediate clinical implications are limited. The evidence was derived entirely from observational studies; the overall certainty of evidence was rated as very low, and considerable heterogeneity remained across study populations, CKD stages, follow-up durations, exposure definitions, and renal outcomes. Consequently, the pooled estimate should not be interpreted as evidence that vitamin D deficiency independently causes CKD progression or that correcting low vitamin D concentrations would prevent deterioration in kidney function.

The stage-stratified findings may nevertheless be clinically informative from a prognostic perspective. Studies involving patients with more advanced CKD showed a stronger association than those involving earlier-stage disease, and studies with longer follow-up also tended to report larger associations. However, these analyses were based on a small number of studies and should be considered exploratory. They do not establish that patients with advanced CKD derive greater renal benefit from vitamin D supplementation, nor do they identify a subgroup in whom treatment specifically for the prevention of CKD progression can currently be recommended.

Similarly, the dose-response analysis suggested progressively higher odds of CKD progression at lower 25(OH)D concentrations, but the certainty of this evidence was low. The analysis relied partly on reconstructed category-level exposure values and cannot establish a clinically actionable threshold. Therefore, the reference value used in the dose-response model should not be interpreted as a therapeutic target, and the present findings do not support the conclusion that maintaining serum 25(OH)D above a specific concentration would reduce the risk of CKD progression.

From a clinical perspective, vitamin D status may therefore be considered a potential prognostic marker rather than an established therapeutic target for CKD progression. Decisions regarding vitamin D assessment or treatment should not be based on the present meta-analysis alone. The key unanswered question is whether low vitamin D status is primarily a marker of disease severity and adverse health status or represents a modifiable factor capable of altering the course of kidney disease. Resolving this distinction will require prospective studies with repeated measurements of vitamin D status and kidney function, together with adequately powered randomized trials evaluating clinically important renal outcomes.

## 5. Conclusion

This systematic review and meta-analysis found that lower serum 25(OH)D concentrations were associated with a higher likelihood of CKD progression. However, the considerable between-study heterogeneity, heterogeneous definitions of renal progression, potential residual confounding and reverse causation, and the very low certainty of the overall evidence substantially limit causal and clinical interpretation. The dose-response findings provide additional evidence of an association but do not establish a therapeutic threshold or demonstrate that increasing vitamin D concentrations would improve renal outcomes.

Future studies should use standardized definitions of CKD progression, adequately account for major confounding factors, and examine changes in vitamin D status and kidney function longitudinally. Ultimately, adequately powered randomized controlled trials are required to determine whether correction of vitamin D deficiency has any effect on clinically important renal outcomes, including sustained eGFR decline, kidney failure, and initiation of kidney replacement therapy. Until such evidence is available, the present findings should be regarded as hypothesis-generating and should not independently guide screening or supplementation strategies for the prevention of CKD progression.

## Author contributions

**Conceptualization:** Sayed Yousef Mojtahedi, Paniz Pourpashang.

**Data curation:** Sayed Yousef Mojtahedi, Paniz Pourpashang.

**Investigation:** Mohsen Sedighiyan, Paniz Pourpashang.

**Methodology:** Sayed Yousef Mojtahedi, Mohsen Sedighiyan.

**Writing – original draft:** Sayed Yousef Mojtahedi, Mohsen Sedighiyan, Paniz Pourpashang.

**Writing – review & editing:** Mohsen Sedighiyan.


