# Supplementary material for: Association between vitamin D deficiency and chronic kidney disease progression: A GRADE-based dose-response systematic review and meta-analysis
Source: Medicine (Baltimore). 2026 Sep 4;105(36):e50522. doi: 10.1097/MD.0000000000050522 (PMC13549525; doi:10.1097/MD.0000000000050522)
Supplement: Supplementary file 1 [file medi-105-e50522-s001.docx]

**Supplementary File 1:** **Details of the Search Strategy Used in Different Databases**

**Search Strategy**

A comprehensive search strategy was developed to identify relevant studies across multiple electronic databases, including PubMed, Scopus, Web of Science, Cochrane Library, and Embase. The search was conducted with no language restrictions but was limited to studies published in English and available from 1990 until November 13, 2024. The strategy combined controlled vocabulary (e.g., Medical Subject Headings [MeSH] in PubMed, Emtree in Embase) and free-text keywords related to chronic kidney disease (CKD), vitamin D deficiency, and progression of renal disease. The following search terms and their synonyms were used:

- **Chronic Kidney Disease**: "chronic kidney disease" OR "CKD" OR "renal impairment" OR "chronic renal failure" OR "glomerular filtration rate" OR "eGFR" OR "end-stage renal disease" OR "ESRD" OR "dialysis" OR "kidney transplantation"
- **Vitamin D**: "vitamin D deficiency" OR "vitamin D insufficiency" OR "25-hydroxyvitamin D" OR "25(OH)D" OR "cholecalciferol" OR "ergocalciferol"
- **Progression**: "progression" OR "decline" OR "worsening" OR "advancement" OR "outcome"

**Database-Specific Search Strings**

1. **PubMed**:
   - ("chronic kidney disease"[MeSH Terms] OR "CKD" OR "renal impairment" OR "glomerular filtration rate"[MeSH Terms] OR "eGFR" OR "end-stage renal disease"[MeSH Terms] OR "ESRD" OR "dialysis"[MeSH Terms] OR "kidney transplantation"[MeSH Terms]) AND ("vitamin D deficiency"[MeSH Terms] OR "25-hydroxyvitamin D"[MeSH Terms] OR "25(OH)D" OR "cholecalciferol"[MeSH Terms] OR "ergocalciferol"[MeSH Terms]) AND ("progression"[All Fields] OR "decline"[All Fields] OR "outcome"[MeSH Terms]) AND ("1990/01/01"[PDAT] : "2024/11/13"[PDAT]) AND English[Language]
2. **Scopus**:
   - TITLE-ABS-KEY("chronic kidney disease" OR "CKD" OR "renal impairment" OR "glomerular filtration rate" OR "eGFR" OR "end-stage renal disease" OR "ESRD" OR "dialysis" OR "kidney transplantation") AND TITLE-ABS-KEY("vitamin D deficiency" OR "25-hydroxyvitamin D" OR "25(OH)D" OR "cholecalciferol" OR "ergocalciferol") AND TITLE-ABS-KEY("progression" OR "decline" OR "outcome") AND PUBYEAR > 1989 AND PUBYEAR < 2025 AND LANGUAGE(english)
3. **Web of Science**:
   - TS=("chronic kidney disease" OR "CKD" OR "renal impairment" OR "glomerular filtration rate" OR "eGFR" OR "end-stage renal disease" OR "ESRD" OR "dialysis" OR "kidney transplantation") AND TS=("vitamin D deficiency" OR "25-hydroxyvitamin D" OR "25(OH)D" OR "cholecalciferol" OR "ergocalciferol") AND TS=("progression" OR "decline" OR "outcome") AND PY=(1990-2024) AND LA=(English)
4. **Cochrane Library**:
   - ("chronic kidney disease" OR "CKD" OR "renal impairment" OR "glomerular filtration rate" OR "eGFR" OR "end-stage renal disease" OR "ESRD" OR "dialysis" OR "kidney transplantation") AND ("vitamin D deficiency" OR "25-hydroxyvitamin D" OR "25(OH)D" OR "cholecalciferol" OR "ergocalciferol") AND ("progression" OR "decline" OR "outcome") [Filters: Published 1990 to 2024, English]
5. **Embase**:
   - ('chronic kidney disease'/exp OR 'CKD' OR 'renal impairment' OR 'glomerular filtration rate'/exp OR 'eGFR' OR 'end-stage renal disease'/exp OR 'ESRD' OR 'dialysis'/exp OR 'kidney transplantation'/exp) AND ('vitamin D deficiency'/exp OR '25-hydroxyvitamin D'/exp OR '25(OH)D' OR 'cholecalciferol'/exp OR 'ergocalciferol'/exp) AND ('progression' OR 'decline' OR 'outcome'/exp) AND [1990-2024]/py AND [English]/lim
